# A Transfer-Based Convolutional Neural Network Model with Multi-Signal Fusion and Hyperparameter Optimization for Pump Fault Diagnosis

**DOI:** 10.3390/s23198207

**Published:** 2023-09-30

**Authors:** Zhigang Zhang, Aimin Tang, Tao Zhang

**Affiliations:** School of Mechanical Engineering, Sichuan University, Chengdu 610065, China; tangam@hhcp.com.cn (A.T.); nic6700@scu.edu.cn (T.Z.)

**Keywords:** pump fault diagnosis, convolutional neural network, transfer learning, multi-signal fusion, hyperparameter optimization

## Abstract

Pumps are one of the core components of drilling equipment, and their fault diagnosis is of great significance. The data-driven approach has made remarkable achievements in the field of pump fault diagnosis; however, most of them are easily affected by complex background conditions and usually suffer from data scarcity problems in real-industrial scenarios, which limit their application in practical engineering. To overcome the above shortcoming, a novel framework for a model named Hyperparameter Optimization Multiple-Signal Fusion Transfer Convolution Neural Network is proposed in this paper. A convolutional neural network model based on transfer learning is built to promote well-learned knowledge transfer over different background conditions, improve robustness, and generalize the model to cross-domain diagnosis tasks. The multi-signal fusion strategy is involved in capturing system state information for establishing the mapping relationship between the raw signal and fault pattern by integrating the multi-physical signal with the weight allocation protocol. The hyperparameter optimization method is explored in conjunction with the transfer-based model by integrating Grid Search with the Gradient Descent algorithm for further improvement of diagnosis performance. Results show that the proposed model can effectively realize the fault diagnosis of pumps under different background conditions, achieving 95% accuracy.

## 1. Introduction

With the increasing demand for natural resources in human society, drilling rigs play a significant role in the extraction process of petroleum and gas resources. The pump is one of the core components of the drilling rig, which is responsible for converting mechanical energy into hydraulic energy and providing hydraulic oil to the drilling rig [1]. During the operation of the pump, chemical corrosion and physical effects such as pressure and friction will cause waste to the parts of the pump, resulting in pump failure. Thus, the fault diagnosis of the pump is of great importance to ensure the continuous work of the drilling dig and the successful extraction of petroleum and gas resources.

At present, research on fault diagnosis has focused on the data-driven approach [2], which diagnoses faults by examining and processing raw signals collected from the system [3]. A data-driven approach does not need to manually extract signal features based on expert knowledge but relies on data support to automatically extract signal features and finally obtain fault diagnosis results. The fault diagnosis of the pump based on the data-driven approach is made up of signal processing methods [4] and artificial intelligence methods [5].

The signal types required for fault diagnosis of the pump based on the signal processing method include vibration signals [6] and sound signals [7]. Usually, the feature extraction methods employ wavelet transformation [8] and fast Fourier transformation [9]. Feature selection methods typically use principal component analysis [10] and independent component analysis [11]. The fault diagnosis of the pump based on the signal processing method presents good performance in noise filtration [12], signal capture [13], and extension optimization [14]. On the other hand, its limitations have an impact on the result of the fault diagnosis. For example, there is randomness in time-domain analysis, which may cause misjudgment in the diagnosis of serious faults and is not suitable for the analysis of strongly fluctuating signals; frequency-domain analysis cannot reflect temporal characteristics and the sensitivity to early fault diagnosis. These factors will affect the fault diagnosis of the pump.

The fault diagnosis method of pumps based on artificial intelligence has achieved long-term development due to the wide application of deep learning. The fully connected neural network models, such as the deep belief network [15], the deep Boltzmann machine [16], and the deep auto encoder [17], have successfully solved plenty of classification problems and been applied to the fault diagnosis of pumps. However, the traditional fully connected neural network model relies on a large number of trainable network parameters to guarantee fault diagnosis performance, which inevitably increases the training burden and affects network convergence speed. Different from that, the convolutional neural network (CNN) [18], which realized feature extraction through the sparse connection of the convolution kernel and weight sharing protocol, can alleviate the above problem. Therefore, some researchers have used convolutional neural network models for pump fault diagnosis in recent years.

For example, Yan et al. [19] proposed a seven-layer CNN model setting method based on the base period, which realized the fault diagnosis of the hydraulic pump. Tang et al. [20] presented a CNN-based hydraulic pump fault diagnosis method that converted vibration signals into image features through continuous wavelet transform and established a new CNN model framework combining feature extraction and classification. Tang et al. [21] proposed a CNN model based on the batch normalization strategy, which extracted the signal features and transformed the synchronous noise of the vibration signal to realize the fault diagnosis of the pump. Tang et al. [22] utilized the continuous wavelet transform to obtain the time-frequency characteristics of the pressure signal and established the CNN model to realize the fault diagnosis of the hydraulic pump.

Although the CNN methods significantly improve performance for pump fault diagnosis, those normal approaches still pose two potential problems that limit their application in practical engineering. More specifically, the background conditions of the pump will inevitably change, which means it is difficult for the established neural network model to generalize the fault pattern knowledge from the labeled training data to the unlabeled testing data due to the interference of variable background conditions. In addition, real industrial scenarios pose challenges to the collection of data, which often leads to the problem of data scarcity. Affected by safety and economic factors, data collection is generally carried out only for a specific period of time, which limits the amount of data. The time distribution of construction machinery in different working states is not average; therefore, the collected data are unbalanced. A complex background environment will also have a negative impact on the quality of the data. Since the quality, distribution, and even quantity of sampled data from different background conditions cannot be guaranteed, this inevitably increases the difficulty of neural network models in cross-domain diagnostic tasks and reduces the usability of the method in real engineering scenarios.

In this condition, the transfer learning strategy, which has the capability to reuse the well-learned knowledge from one condition to another by fine-tuning operations, is beneficial for alleviating the above problem. From the perspective of background conditions, due to the advanced learning of common knowledge in different backgrounds, the impact of background changes is only caused by characteristic knowledge, and the sensitivity of neural network models to background conditions is significantly reduced. From the perspective of data scarcity, due to the repeated use of knowledge across conditions, high-quality data under certain conditions can provide support for learning tasks under all conditions, thereby reducing the dependence of neural network models on large amounts of training data. Therefore, the transfer learning strategy can reduce the learning cost by reusing the knowledge obtained from the source task in the training of the target task. The reduction of the learning cost makes the transfer learning strategy available on an edge device with limited resources, and many researchers have involved this technology in fault diagnosis fields.

Nevertheless, to the best of our knowledge, no research has been introduced for pump fault diagnosis in a real-industrial scenario so far. However, similar research based on transfer learning for rotating machine fault diagnosis tasks has been widespread in recent years, which may provide an effective reference for fault pattern detection of pumps in real-industrial environments. For example, Tang et al. [23] proposed a CNN model of fault diagnosis based on transfer learning. The method processes the vibration signal of rolling bearings by Fourier transform, obtains the data set of the source domain and target domain, and trains the CNN model to realize fault diagnosis. Zhang et al. [24] froze all layers except the last layer of the trained CNN model in the source domain with single-type data. And then a small amount of target domain data was used to train the network parameters of the last layer to realize the transfer-based fault diagnosis of rolling bearings. Shao et al. [25] made use of the network parameters of the trained CNN model to take the place of the randomly initialized network parameters, realized transfer, and completed the classification task supported by single-type data of the target domain by changing the number and weight of the network parameters. Zhao et al. [26] suggested that different convolution kernels of scale could be used to extract different features of vibration signals and proposed a multi-scale CNN transfer learning model framework for fault diagnosis of rolling bearings.

Although the current transfer learning models can improve diagnosis performance to some extent, most of them only rely on a single physical signal. Compared with the multiple types of physical signals, a single physical signal may not be able to fully describe the complex working state of a machine such as a pump. In this way, the extracted feature from the collected single physical signal cannot form sufficient information representation to make it susceptible to random factors, which limits the robustness and generalization of the transfer learning model for fault diagnosis under variable background conditions.

In addition, the hyperparameter setting of the transfer learning model is an important problem, and the proper combination of hyperparameters can improve the performance of the transfer learning model. The manual setting of hyperparameters will be affected by the operator experience, and the number of attempts is relatively limited; therefore, it is not easy to obtain the optimal combination of hyperparameters. Compared with manually setting the hyperparameters of the transfer learning model, automatic tuning of the hyperparameter combination presents great advantages [27]. The optimization algorithm [28] was used to optimize the hyperparameter combination to reduce the influence of randomness on the setting of hyperparameters and improve the performance of the transfer learning model.

To solve these problems, a novel framework for a model named Hyperparameter Optimization Multi-Signal Fusion Transfer Convolution Neural Network (OMTCNN) is proposed in this paper. A new transfer-based convolutional neural network model is first designed to promote well-learned knowledge transfer over different background conditions for improving robustness and generalizing the model in cross-domain diagnosis tasks. Then, the multi-signal fusion strategy is involved to capture the state information of the pump for establishing the mapping relationship between the raw signal and fault pattern by integrating the multi-physical signal with the weight allocation protocol. Afterward, a hyperparameter optimization method is explored to arm with the transfer learning model by integrating Grid Search with the Gradient Descent algorithm for further improving diagnosis performance. By doing this, the OMTCNN model can effectively realize the fault diagnosis of pumps under different background conditions.

The main contributions of the network model proposed in this paper are as follows:

- Strengthen the ability of convolutional neural networks to diagnose pump faults under different background conditions by introducing transfer learning.
- Break through the limitation of a single type of signal for pump fault diagnosis by using multiple types of signals for multi-signal fusion.
- Optimize the fault diagnosis results of the pump through the automatic setting of hyperparameters.

The remainder of this article is organized as: Section 2 introduces the basic theory of convolutional neural networks and transfer learning. Section 3 describes a convolutional neural network model based on transfer learning, a multi-signal fusion module, a hyperparameter optimization module, and the OMTCNN model. Section 4 presents the design of the experiment using the relevant network model and analyzes the experiment results. Section 5 states the conclusions about the OMTCNN model.

## 2. Theory

### 2.1. Convolutional Neural Network

A convolutional neural network [29] is a feedforward neural network with convolutional operations and a deep structure. It is mainly composed of a convolutional layer, a pooling layer, a full connection layer, an activation function, and a Dropout.

The convolutional layer consists of neurons with learnable weights and bias constants, which are used to convolute the input data to realize feature extraction. The convolution operation can be expressed as follows:

(1) $$\left(f*g\right)\left(x,y\right)=\sum_{i=1}^{m}\sum_{j=1}^{n}f\left(i,j\right)g\left(x-i,y-j\right)$$

where f represents the input data, g stands for the convolution kernel, m and n respectively represent the width and height of the convolution kernel, and are $\left(x,y\right)$ the coordinates of a data value in the output feature map.

The convolution operation mainly involves two important concepts, namely, sparse connection and weight sharing. Sparse connection refers to the fact that the input data and the convolution layer are not fully connected but create connections in the local area of the convolution layer, realizing the extraction of the input data features through the local perception of the convolution kernel. The weight sharing obeys the assumption that the importance characterization results of input data features from the same dimensional plane should be consistent. In other words, the weights and bias constants of the same convolution kernel remain constant. Weight sharing can effectively reduce the number of network training parameters by means of convolution kernel check feature repeated recognition, without considering the feature location distribution.

The pooling layer aggregates the input data region into the output of the feature map through the region adjacency of the feature mapping, which is used to describe the association between the output and the region, and realizes the down-sampling processing of the input data. Down-sampling processing can improve network efficiency and avoid the overfitting phenomenon. The pooling layer is also used to reduce the sensitivity of the convolutional layer to the target position so that its feature extraction is not seriously affected by the change in the target position. The pooling layer generally adopts the maximum pooling strategy or the average pooling strategy, which can be stated by the following formula:

(2) $$X_{i,j}=\underset{p=i}{max}\left(i+k-1\right)\underset{q=j}{max}\left(j+k-1\right)Z_{p,q}$$

(3) $$X_{i,j}=\frac{1}{k^{2}}\sum_{p=i}^{i+k-1}\sum_{q=j}^{j+k-1}Z_{p,q}$$

where Z represents the input feature mapping, k stands for the size of the pooling window, and X is called the output feature mapping.

The fully connected layer maps the features extracted from the convolution layer and the pooling layer to the sample space. These features can be obtained by the convolutional layer and the pooling layer mapping the input data to the feature space. Ignoring the influence of spatial structural features after feature flattening, combining all local features of input data into global features, and achieving the representation of input data features by output.

The activation function is a nonlinear function that acts on the output of each neuron in the convolutional layer and the fully connected layer. The activation function introduces non-linear factors to the network so that it can approach almost arbitrary functions and improve its expression ability.

Dropout is a regularization method that randomly and temporarily discards some neuronal nodes with a certain probability in network training. It is mainly used to solve the overfitting problem that the accuracy of the network is high in the training set and low in the testing set, so as to improve the stability of the network.

Convolutional neural networks are trained through forward propagation of input data, back propagation of the loss function representing output errors, and updating of network parameters. The forward propagation calculation can be expressed by the following formula:

(4) $$a^{\left(l\right)}=g\left(z^{\left(l\right)}\right)$$

(5) $$z^{\left(l+1\right)}=W^{\left(l\right)}a^{\left(l\right)}+b^{\left(l\right)}$$

where $a^{\left(l\right)}$ and $z^{\left(l\right)}$ respectively represent the activation function value and the input weight of layer l, g is the activation function, $W^{\left(l\right)}$ and $b^{\left(l\right)}$ respectively stand for the weights and bias of layer l. The back propagation calculation can be expressed as follows:

(6) $$\delta^{\left(L\right)}={▽}_{a}J⊙g^{\prime }\left(z^{\left(L\right)}\right)$$

(7) $$\delta^{\left(l\right)}=({\left(W^{\left(l\right)}\right)}^{T}\delta^{\left(l+1\right)})⊙g^{\prime }(z^{\left(l\right)})$$

(8) $$\frac{\partial J}{\partial W^{\left(l\right)}}=\delta^{\left(l+1\right)}{\left(a^{\left(l\right)}\right)}^{T}$$

(9) $$\frac{\partial J}{\partial b^{\left(l\right)}}=\delta^{\left(l+1\right)}$$

where $\delta^{\left(l\right)}$ is the error in layer l, ${▽}_{a}J$ is the partial derivative of the Loss function on the output result, and ⊙ represents the Hadamard product, that is, multiplication by element, $g^{\prime }$ stands for the derivative of the activation function, $W^{\left(l\right)}$ and $b^{\left(l\right)}$ respectively stands for the weights and bias of layer l. The convolutional neural network gradually converges the Loss function output to the local minimum through gradient descent, i.e., to complete the training of the convolutional neural network. The trained convolutional neural network can realize fault diagnosis.

### 2.2. Transfer Learning

Transfer learning [30] is a method of learning a new task by transferring knowledge from a learned task. The method uses the knowledge of related learning tasks in the source domain, composed of feature space and edge probability distribution, to support the learning of related tasks in the target domain, also composed of feature space and edge probability distribution.

Domain, which can be denoted as $Ɗ=\left\{Ӽ,\;P\left(X\right)\right\}$, consists of two components: a feature space Ӽ and a marginal probability distribution $P\left(X\right)$, where $X=\{x|x_{i}\;\in \;Ӽ,\;i=1,\cdot \cdot \cdot ,\;N\}$ is a dataset that contains N instances.

Task can be denoted as $Ƭ=\left\{Ƴ,\;f\left(\cdot \right)\right\}$ when giving a specific domain Ɗ, consists of two components: a label space Ƴ and a mapping function $f\left(\cdot \right)$, where $Y=\{y|y_{i}\;\in \;Ƴ,\;i=1,\cdot \cdot \cdot ,\;N\}$ is a label set for the corresponding instances in Ɗ. The mapping function $f\left(\cdot \right)$, also denoted as $f\left(x\right)=P(y|x)$ is a non-linear and implicit function that can bridge the relationship between the input instance and the predicted decision, which is expectedly learned from the given datasets.

Transfer Learning, given a source domain ${Ɗ}^{S}=\left\{{Ӽ}^{S},\;P^{S}\left(X^{S}\right)\right\}$ with the source task ${Ƭ}^{S}=\left\{{Ƴ}^{S},\;f^{S}\left(\cdot \right)\right\}$ and a target domain ${Ɗ}^{T}=\left\{{Ӽ}^{T},\;P^{T}\left(X^{T}\right)\right\}$ with the target task ${Ƭ}^{T}=\left\{{Ƴ}^{T},\;f^{T}\left(\cdot \right)\right\}$, aims to learn a better mapping function $f^{T}\left(\cdot \right)$ for the target task ${Ƭ}^{T}$ with the transferable knowledge gained from the source domain ${Ɗ}^{S}$ and task ${Ƭ}^{S}$.

The high-dimensional connection obtained by the convolutional neural network model in the learning task in a specific background condition, that is, the mapping of the source domain features to the sample space, has a guiding effect on the relevant learning task in other background conditions. The knowledge obtained in the source domain learning task can be used to help the learning of the corresponding task in the target domain by using transfer learning. This reduces the interference of background condition changes in the convolutional neural network model. From the perspective of data volume, since the convolutional neural network model obtains the common knowledge of the relevant learning tasks of the source domain in advance of the learning tasks of the target domain, the learning of the relevant tasks of the target domain mainly focuses on the unique knowledge related to the background condition, and the demand for the amount of data will be relatively reduced. Therefore, a convolutional neural network model based on transfer learning overcomes the shortcomings of the traditional convolutional neural network model.

In transfer learning, different techniques are applied to convolutional neural network models, and typically they are a specific combination of pre-training, freezing, fine-tuning, and adding new layers. A network model trained with source domain data are called a pretrained network model, consisting of pretrained layers. Freeze and fine-tuning are techniques that use some or all layers of pre-trained network models to train on a target domain. Freezing certain layers means that the trainable parameters do not change and are constant for frozen layers in pre-trained network models. Fine-tuning means initializing trainable parameters with pre-trained layers rather than randomly initializing the entire network model or some selected layers. Another new technique is based on freezing a pre-trained network model and adding a new layer to that model to train on target data.

The strategy of freezing the first several layers of the convolutional neural network model can be used to carry out transfer learning, which can further demonstrate the advantages of the convolutional neural network model based on transfer learning. According to the characteristics that convolutional neural networks extract low-dimensional features with shallow layers from data and convolutional neural networks extract high-dimensional features with deep layers from data, the strategy of freezing the shallow layers of convolutional neural networks can be used to carry out transfer learning. The common knowledge of the learning task under different background conditions is learned by the shallow layer of the convolutional neural network, and the common knowledge is fixed by freezing the shallow layer of the convolutional neural network. The characteristic knowledge of learning tasks under different background conditions is learned through the deep layer of the convolutional neural network, and the frozen shallow convolutional neural network is carried out through transfer learning to complete the effective extraction of high-dimensional features of data. Furthermore, because the convolutional neural network after transfer learning mainly extracts high-dimensional features of data, the demand for data volume will be relatively reduced. Therefore, the pump fault diagnosis results of the convolutional neural network model based on transfer learning under variable background conditions have better performance than the traditional convolutional neural network model.

## 3. Method

### 3.1. Multi-Signal Fusion Module

**Figure 1:**
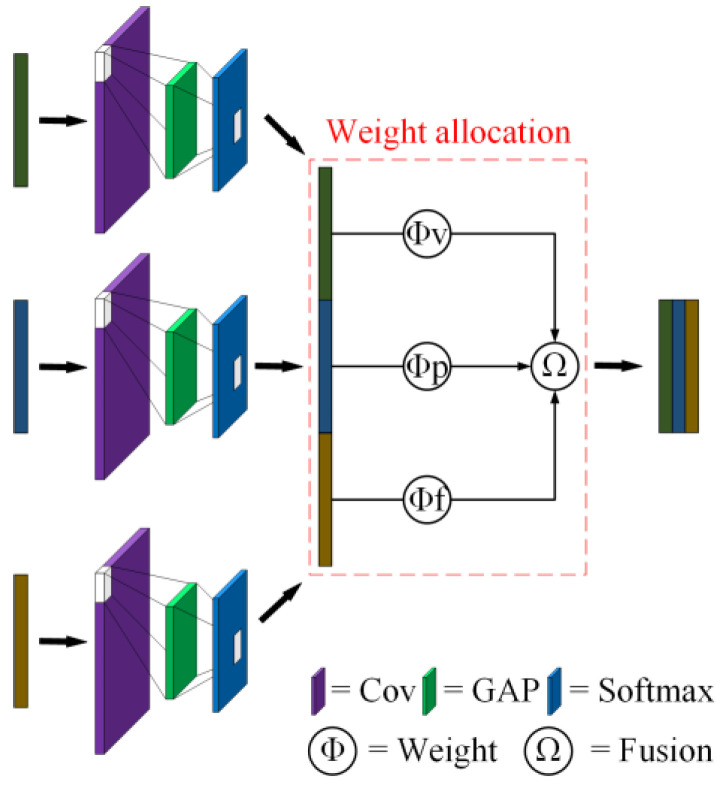
Structure diagram of multi-signal fusion module.

In the process of data collection, multiple types of signals can be collected by installing various types of sensors. Compared with a single type of signal, multiple types of signals can describe the pump system from different angles, which can better reflect the essential characteristics of the pump system, increase resistance to random factors, and improve robustness. However, traditional convolutional neural network models are not good at directly processing data of multiple signal types, and the effect of pump fault diagnosis is limited. In order to solve this problem, a multi-signal fusion module based on weight allocation is proposed in this paper. The structural diagram of the multi-signal fusion module is shown in Figure 1.

The multi-signal fusion module based on weight allocation mainly includes three parts: standard normalization, reliability operation, and weight setting. The module is used to fuse a group of signal samples of multiple types into a fusion sample containing multi-signal-type information. The standard normalization part is responsible for adjusting the probability distribution of multiple types of signal samples to the same statistical space for subsequent calculations. In the reliability operation part, the reliability degree of each signal sample of different types is obtained by means of convolution operation, global average pooling operation, and classification operation. The weight-setting section makes weight adjustments according to the reliability of each signal sample of different types. Finally, the multi-signal fusion module combines a group of signal samples of multiple types after weight setting into a fusion sample containing multi-signal type information. The fusion sample of multiple signal types can characterize the pump system more appropriately and accurately than the original signal of multiple types. Therefore, the multi-signal fusion module based on weight allocation can realize the pump fault diagnosis of multiple signal types.

Different types of sensors can collect different types of signals to reflect the operating condition of the pump, such as vibration signals [31], pressure signals [32], flow signals [33], etc. When the pump fails, it is often accompanied by abnormal vibration, shock noise, and flow reduction; therefore, physical quantities such as vibration, pressure, and flow can be used as the basis for the fault diagnosis of the pump. Thus, in this paper, vibration sensors, pressure sensors, and flow sensors are utilized to acquire relevant types of signals from the pump.

The A/D converter converts multiple types of signals into a data matrix that characterizes the pump’s operating state from different angles, including the vibration data matrix, pressure data matrix, and flow data matrix. The multi-signal fusion module based on weight allocation is used to process the data matrix.

Firstly, the numerical distribution of the vibration data matrix, pressure data matrix, and flow data matrix is adjusted by standard normalization as follows:

(10) $$x_{v}^{*}=\frac{x_{v}-\mu_{v}}{\delta_{v}}$$

(11) $$x_{p}^{*}=\frac{x_{p}-\mu_{p}}{\delta_{p}}$$

(12) $$x_{f}^{*}=\frac{x_{f}-\mu_{f}}{\delta_{f}}$$

where $x_{\varphi }$ represents the corresponding sample data, $\mu_{\varphi }$ stands for the corresponding sample data mean and $\delta_{\varphi }$ is called the corresponding sample data standard deviation. φ represents v, p and f. So that the numerical distribution of the data matrix of the three signal types is located in the same statistical space, which facilitates the subsequent operation.

Secondly, the reliability of the vibration data matrix, pressure data matrix, and flow data matrix are verified, respectively. After the data matrix passes through the convolution layer, the global average pooling layer, and the Softmax classifier, The calculation process for Softmax is as follows:

(13) $$Softmax\left(z\right)=\frac{e^{z_{i}}}{\sum_{j=0}^{k}e^{z_{j}}}$$

where z represents the input vector, $e^{z_{i}}$ is the standard exponential function for the input, k is the number of classes and $e^{z_{j}}$ refers to the standard exponential function for the output. The reliability probability of the vibration data matrix, pressure data matrix, and flow data matrix are obtained, respectively, record as $Rx_{v}$, $Rx_{p}$ and recorded as $Rx_{f}$.

Finally, according to the relative value of the reliability probability of the vibration data matrix, pressure data matrix, and flow data matrix, the respective weight ratio is obtained as follows:

(14) $$W_{x_{v}}=\frac{Rx_{v}}{Rx_{v}+Rx_{p}+Rx_{f}}\times 100\%$$

(15) $$W_{x_{p}}=\frac{Rx_{p}}{Rx_{v}+Rx_{p}+Rx_{f}}\times 100\%$$

(16) $$W_{x_{f}}=\frac{Rx_{f}}{Rx_{v}+Rx_{p}+Rx_{f}}\times 100\%$$

The vibration data matrix, pressure data matrix, and flow data matrix are weighted and fused into a fusion matrix. And match the corresponding Loss function:

(17) $$Loss=W_{x_{v}}Loss_{x_{v}}+W_{x_{p}}Loss_{x_{p}}+W_{x_{f}}Loss_{x_{f}}$$

The multi-signal fusion module based on weight allocation improves the input quality and reverse update of the convolutional neural network model based on transfer learning, which has a positive effect on the fault diagnosis of pumps.

### 3.2. Convolutional Neural Network Model Based on Transfer Learning

**Figure 2:**
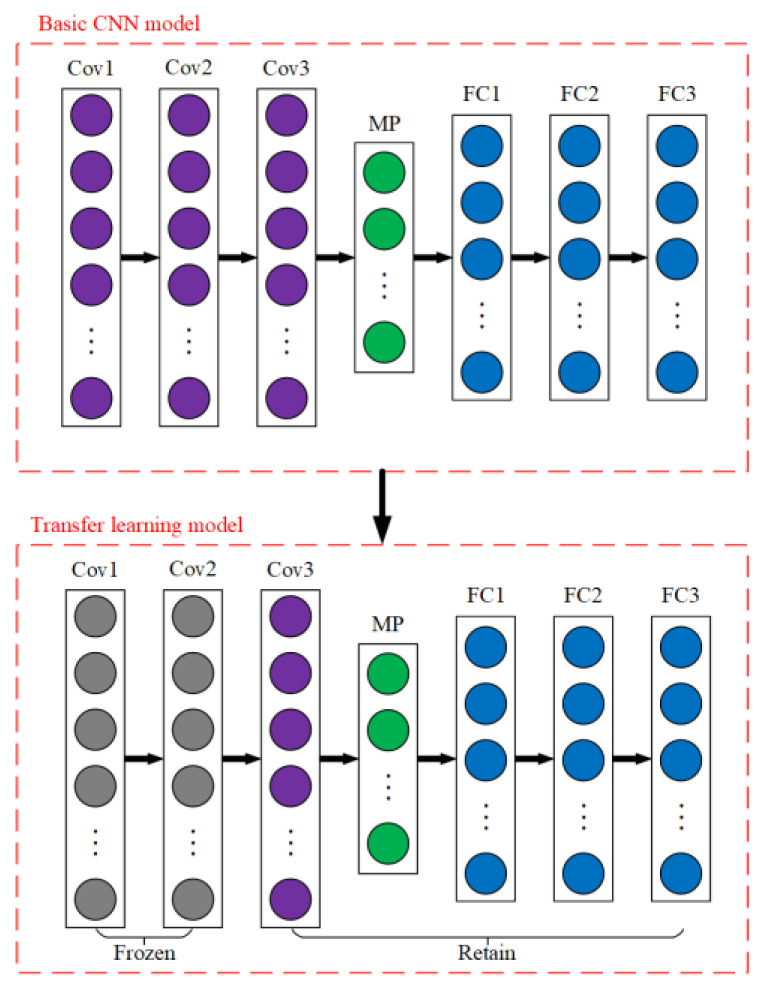
Structure diagram of a convolutional neural network model based on transfer learning.

When the traditional convolutional neural network model solves the problem of pump fault diagnosis under different background conditions, the fault diagnosis accuracy of the traditional convolutional neural network model is relatively limited due to the influence of background condition changes and the fact that the amount of data in all background conditions may not be sufficient. To solve the problem, it is proposed to develop a convolutional neural network model based on transfer learning, which takes advantage of transfer learning to solve the pump fault diagnosis of a convolutional neural network model under variable background conditions. The structural diagram of the convolutional neural network model based on transfer learning is shown in Figure 2.

The basic convolutional neural network model consists of three convolutional layers, a maximum pooling layer, and three fully connected layers, and sets the ReLU activation function and Dropout.

The application of transfer learning in convolutional neural networks involves a variety of frozen strategies. For example, freezing all layers except the last layer of the pre-trained network for the dataset in the source domain, no longer updating the training network parameters of the frozen layer, and training the final layer of the pretrained network using the dataset in the target domain may also freeze some layers of the pre-trained network for the dataset in the source domain or not to perform freezing operations, and use the dataset in the target domain to train some or all layers of the pre-trained network.

In this paper, the convolutional neural network model based on transfer learning is used to diagnose pump faults under different background conditions. Firstly, a relatively suitable background condition is selected as the source domain, and the data under this condition is used to train the convolutional neural network model. Secondly, the first two layers of the trained convolutional neural network model are frozen. Finally, the first two layers of a frozen convolutional neural network model are used to carry out fault diagnosis under other background conditions, that is, the target domain, and realize transfer learning under different background conditions. The convolutional neural network model based on transfer learning can realize transfer learning under any background condition, so as to realize pump fault diagnosis under variable background conditions.

### 3.3. Hyperparameter Optimization Module

The setting of hyperparameters will affect the performance of the convolutional neural network model, and a reasonable setting of hyperparameters can effectively improve the accuracy of fault diagnosis in the convolutional neural network model. The method of manually setting hyperparameters is easily dictated by empirical factors; therefore, the difference between the experimenters who set hyperparameters may affect the result of the fault diagnosis. Moreover, due to the limited efficiency of manually setting the hyperparameters, it is often impossible to obtain a relatively appropriate combination of hyperparameters. To solve this problem, a hyperparameter optimization module based on the algorithm integrating Grid Search with Gradient Descent is proposed in this paper.

The relatively important hyperparameters of the convolutional neural network model mainly include batch size and learning rate. The hyperparameter optimization module can be used to find the optimal combination of batch size and learning rate within a certain range. For the batch size, its distribution is discrete, and the optimal selection is usually concentrated on partial values; therefore, the grid search method can be used to find the most optimal batch size. As for the learning rate, its distribution is continuous and the minimum value is few; therefore, the method of gradient descent can be used to find the optimal choice of learning rate. The hyperparameter optimization module reduces the impact of the differences between the experimenters on the results of fault diagnosis and can find a set of relatively suitable hyperparameter combinations in the two-dimensional space composed of batch size and learning rate. Therefore, the hyperparameter optimization module based on the algorithm integrating Grid Search with Gradient Descent can optimize pump fault diagnosis results.

The following methods can be used to select relatively suitable hyperparameter combinations: According to the optimal distribution characteristics of batch size, an optimization range is determined for batch size, and an appropriate value is found in the optimization range to assign a hyperparameter. The optimization range of batch size is composed of discrete values that fit the grid search. It can be represented as follows:

(18) $$B=\left\{2^{0},\;2^{1},\cdot \cdot \cdot ,\;2^{n}\right\}\;n=10$$

and the optimization range of the learning rate is composed of continuous values fit gradient descent as follows:

(19) $$\theta =\theta_{0}-\alpha ▽J\left(\theta_{0}\right)$$

where α is the updating rate of gradient descent, $▽J\left(\theta_{0}\right)$ represents gradient of Loss function $J\left(\theta_{0}\right)$ on parameters $\theta_{0}$.

**Figure 3:**
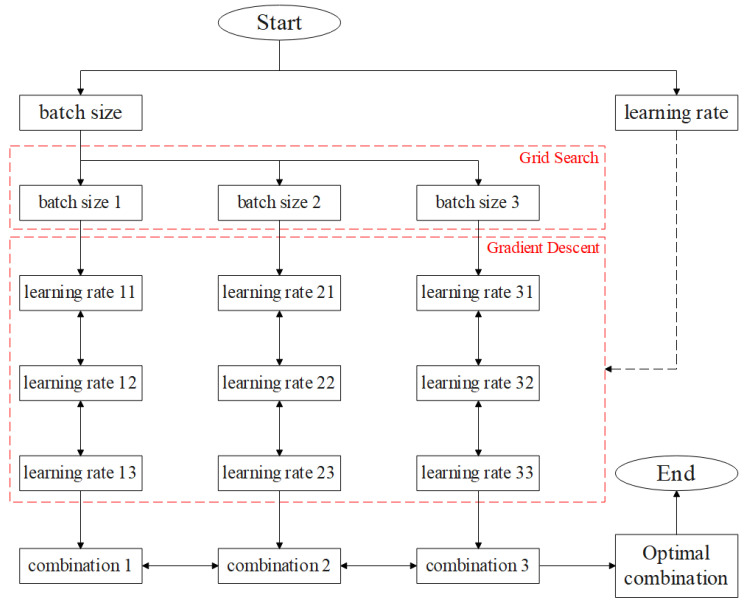
Block diagram of the hyperparameter optimization module.

In the two-dimensional space composed of batch size optimization ranges and learning rate optimization ranges, the algorithm integrates Grid Search with Gradient Descent. That is, the value in the optimization range of a batch size is fixed, the gradient descent is carried out in the optimization range of the learning rate, and then the operation is traversed through all the values in the optimization range of the batch size. In order to increase the probability of finding the optimal value, multiple gradient descents can be performed by random means of multiple initial values. In addition, a break-out mechanism is designed for the hyperparameter optimization module. When the output accuracy of the convolutional neural network model does not reach a new high after several iterations, the iteration is interrupted, and then other numerical combinations are verified. By comparing the combinations of values in the optimization space, a set of relatively suitable values is obtained to assign hyperparameters. The block diagram of the hyperparameter optimization module is shown in Figure 3.

The main optimization process of the hyperparameter optimization module is as follows:

Step 1. Running the Grid Search algorithm within the preset range of batch size yields several batch size values.

Step 2. For each batch size, run the Gradient Descent algorithm multiple times within the preset range of the learning rate to obtain several learning rate values.

Step 3. Several combinations composed of specific batch sizes and corresponding learning rates to obtain the local optimal combination.

Step 4. Screen out the global optimal combination from all the local optimal combinations.

The hyperparameter optimization module based on the algorithm integrating Grid Search with Gradient Descent can automatically set relatively appropriate hyperparameters for the convolutional neural network model based on transfer learning, which provides important help for the fault diagnosis of the pump.

### 3.4. OMTCNN Model

**Figure 4:**
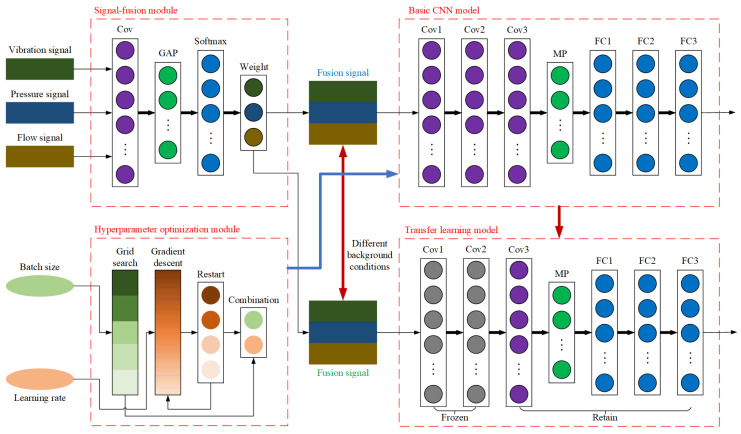
Structure diagram of OMTCNN model.

The model of OMTCNN is presented in Figure 4. It is made up of a convolutional neural network model based on transfer learning, a multi-signal fusion module, and a hyperparameter optimization module.

**Figure 5:**
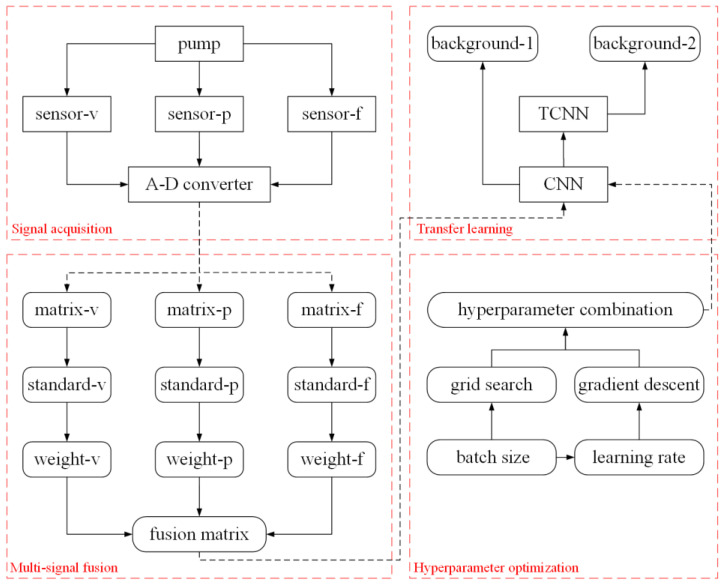
Flow diagram of the OMTCNN model.

The convolutional neural network model based on transfer learning is mainly employed for realizing the transfer training of the convolutional neural network model under different background conditions. The multi-signal fusion module is mainly applied for the data matrix fusion of vibration data matrix, pressure data matrix, and flow data matrix. The hyperparameter optimization module is mainly used for the automatic setting of batch size hyperparameters and learning rate hyperparameters. The operational process of the OMTCNN model is shown in Figure 5.

The main operational process of the OMTCNN model in fault diagnosis is as follows:

Step 1. Obtain the fusion data matrix from the original data through the multi-signal fusion module.

Step 2. Through the hyperparameter optimization module, automatically set hyperparameters for the convolutional neural network model based on transfer learning.

Step 3. The convolutional neural network model based on transfer learning obtains fault diagnosis results in the source domain.

Step 4. Put the fusion data matrix of the target domain into a convolutional neural network model based on transfer learning.

Step 5. The convolutional neural network model based on transfer learning performs transfer learning to obtain fault diagnosis results in the target domain.

## 4. Experiment

### 4.1. Set Up

The pump consists of five cylinders, each containing two valve bodies, namely the suction valve and the release valve. This paper makes four classifications on the fault status of the cylinder according to the failure of the suction valve and the release valve, and the four types of fault status are normal double valve, suction valve failure, release valve failure, and whole valve failure. These four types of fault status correspond here to N, S, R, and W.

**Figure 6:**
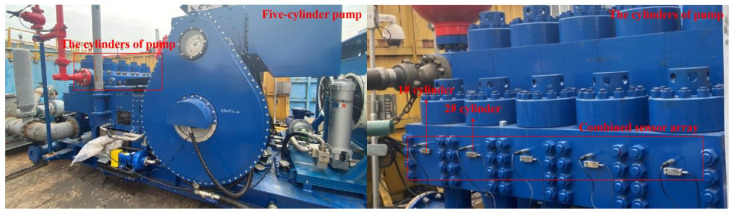
The site of signal collection.

During the operation of the pump, the background conditions, such as the stroke of the pump and the pressure of the pump, will change. The stroke of the pump is mainly maintained at 90 strokes per minute (SPM) and 110 SPM, and the pressure of the pump is mainly maintained at 20 Mpa and 40 Mpa. The conditions of the stroke of the pump and the pressure of the pump can be divided into four situations: 90-20, 90-40, 110-20, and 110-40. In this paper, these four situations correspond to A, B, C, and D conditions. Vibration sensors, pressure sensors, and flow sensors are used to collect the relevant signals from the 1# cylinder and the 2# cylinder of the pump under A, B, C, and D conditions. The signal collection of the five-cylinder pump on site is shown in Figure 6.

The vibration signal, pressure signal, and flow signal are converted by an A/D converter to obtain vibration, pressure, and flow data. Specifically, the sampling frequency of the three types of sensors is 1000 Hz, and the three types of data generated every 1 s is intercepted as a sample. 100 samples with vibration, pressure, and flow data can be obtained each under A, B, C, and D conditions in a 1# cylinder and a 2# cylinder. The vibration data samples, pressure data samples, and flow data samples under specific conditions are arranged according to the collection sequence. Then, a single sample of vibration data, pressure data, and flow data with the same sequence is selected for fusion operation in sequence, and several fusion samples corresponding to the sequence are obtained. Each fusion sample contains vibration, pressure, and flow information. All fusion samples under a specific condition are randomly allocated to form a training set with a proportion of 0.7 and a test set with a proportion of 0.3 and are extended to all conditions to complete the data preparation.

The following experiments were conducted under the Linux-based Ubuntu operating system using the Python programming language in the PyTorch integrated development environment.

### 4.2. Performance Evaluation of Basic CNN Model

In order to evaluate the benchmark performance of the basic CNN model, as shown in Figure 1 above, this experiment employs the basic CNN model for fault diagnosis at A, B, C, and D conditions of a 1# cylinder and a 2# cylinder, respectively. The parameter configuration of the basic CNN model is shown in Table 1.

The accuracy of using the basic CNN model for a 1# cylinder and a 2# cylinder under A, B, C, and D conditions is shown in Table 2.

**Figure 7:**
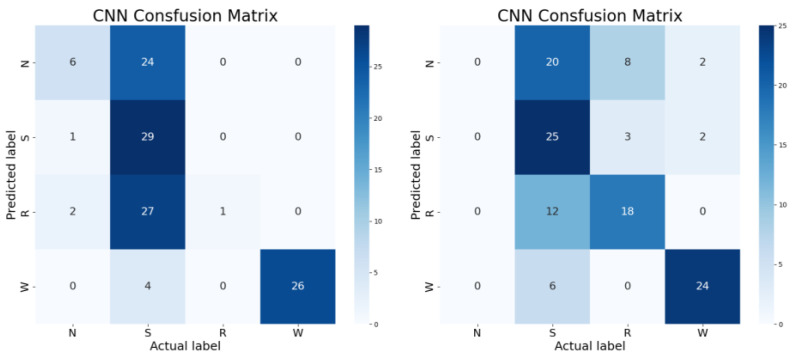

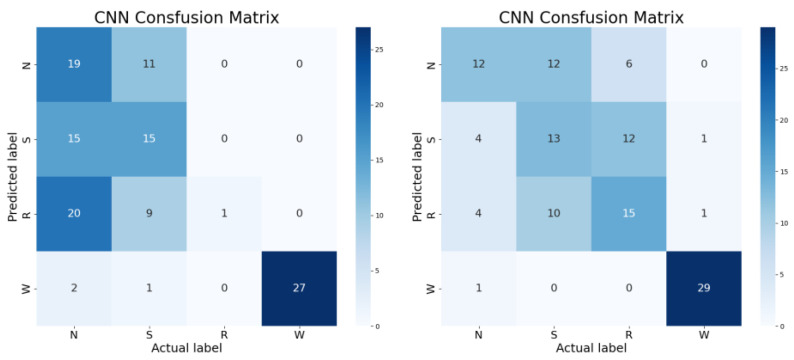
The confusion matrix of the basic CNN model in 1#A, 1#D, 2#B and 2#C.

From Table 2, it can be observed that the fault diagnosis accuracy under A, B, C, and D conditions of the 1# cylinder and 2# cylinder of the basic CNN model is relatively limited, and there are certain fluctuations under A, B, C, and D conditions. Further observation of the confusion matrix of the basic CNN model in 1#A, 1#D, 2#B, and 2#C conditions shown in Figure 7 with types of fault status, namely N, S, R, and W, besides each fault status contains 30 samples.

From Figure 7, it can be observed that the basic CNN model has relatively high fault diagnosis accuracy for type W. However, for other types of fault status, such as N, S, and R, the basic CNN model cannot effectively diagnose them. The experiment result indicates that the basic CNN model has limited accuracy in fault diagnosis and significant differences in sensitivity to different types of fault status. After that, attempt to strengthen the ability of the basic CNN model to diagnose faults under different background conditions by introducing transfer learning.

### 4.3. Performance Analysis of Transfer Convolutional Neural Network Model

In order to demonstrate the improvement of performance through the introduction of transfer learning, this experiment uses the transfer convolutional neural network (TCNN) model to diagnose faults within the 1# cylinder and 2# cylinder, respectively, under the conditions of A, B, C, and D, with any background condition as the source domain and other background conditions as the target domain. The TCNN model employs the transfer learning function of the basis CNN model, and its parameter configuration is consistent with the basis CNN model. The accuracy of using the TCNN model for 1# cylinder and 2# cylinder under the conditions of A, B, C, and D is shown in Table 3 and Table 4.

It can be observed that the TCNN model has a significant improvement in fault diagnosis accuracy compared to the basic CNN model under A, B, C, and D conditions for internal transfer of the 1# cylinder and 2# cylinder, and the fluctuations under A, B, C, and D conditions have been weakened. In further experimentation, the TCNN model performed fault diagnosis on the A, B, C, and D conditions between the 1# cylinder and the 2# cylinder. Through transfer learning, using any background condition as the source domain and other background conditions as the target domain, the experimental results are shown in Table 5 and Table 6.

It can be observed that the fault diagnosis accuracy of the TCNN model in the transfer between the 1# cylinder and the 2# cylinder under A, B, C, and D conditions is generally lower than the accuracy of the internal transfer of the cylinder; however, it still has a certain improvement compared to the basic CNN model and controls the waves under A, B, C, and D conditions. The experiment result indicates that, compared with the basic CNN model, the TCNN model improves generalization ability through transfer learning and maintains a certain degree of robustness. Next, attempt to improve the TCNN model’s processing capacity for multiple types of signals by introducing a multi-signal fusion module.

### 4.4. Comparison of Multi-Signal Fusion Transfer Convolution Neural Network Model and Other Methods

In order to test the performance improvement brought by processing multiple types of signals in the multi-signal fusion module, this experiment uses the multi-signal fusion transfer convolution neural network (MTCNN) model to perform fault diagnosis using transfer learning under the conditions of A, B, C, and D within the 1# cylinder, with any background condition as the source domain and other background conditions as the target domain. The MTCNN model has added a multi-signal fusion module on top of the TCNN model, and its parameter configuration is inherited. Select transfer network models with a structure scale similar to the proposed method are shown in Table 7.

**Figure 8:**
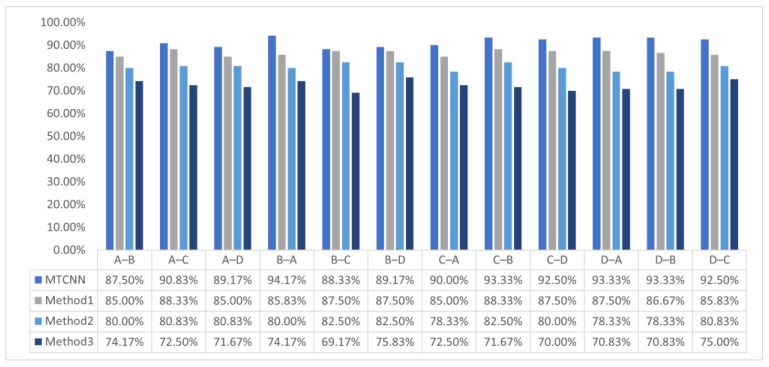
The accuracy of MTCNN and other methods under different background conditions in 1#.

In addition, based on the same or similar model structure and parameter configuration as the related transfer network model, using the same dataset for the experiment is shown in Figure 8.

**Figure 9:**
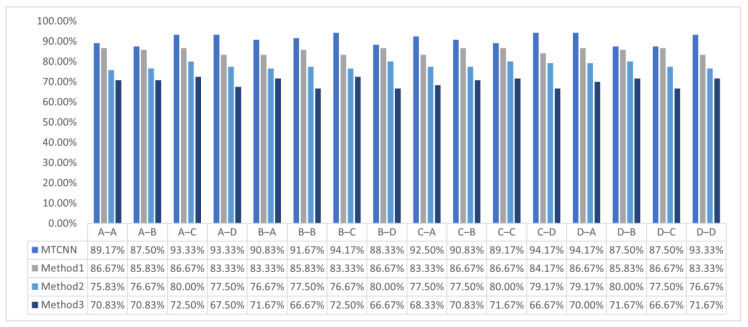
The accuracy of MTCNN and other methods under different background conditions ranges from 2# transfer to 1#.

It can be observed that the MTCNN model has certain advantages in fault diagnosis accuracy compared to other transfer network models in the internal transfer of the 1# cylinder under A, B, C, and D conditions. Further experimentation uses the MTCNN model through transfer learning to diagnose faults in the A, B, C, and D conditions of the 1# cylinder, also using any background condition as the source domain in the 2# cylinder and other background conditions as the target domain in the 1# cylinder. And compare it with other transfer network models shown in Figure 9.

It can be observed that the MTCNN model still has advantages in fault diagnosis accuracy compared to other methods under A, B, C, and D conditions when transferring from a 2# cylinder to a 1# cylinder. However, computation efficiency is also an important indicator for measuring the performance of network models. The computation efficiency of the MTCNN model and other transfer network models on the specific dataset is shown in Table 8.

**Figure 10:**
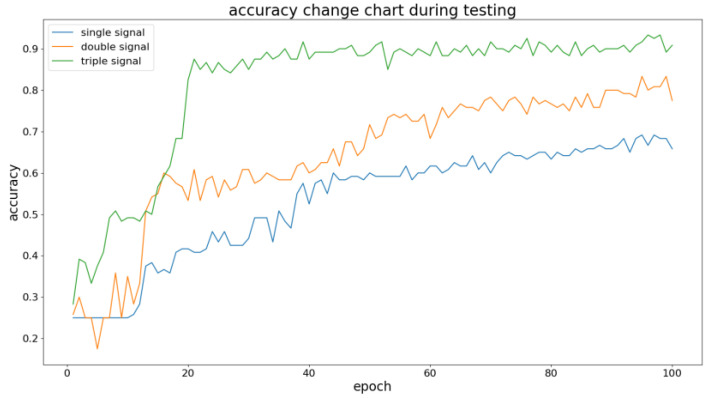
The accuracy of MTCNN under different amount types of signals.

It can be observed that, compared with other methods, the MTCNN model ensures computation efficiency in the process of fault diagnosis. And if only considering the fault diagnosis results of the internal transfer of 1# cylinder and the transfer from 2# cylinder to 1# cylinder under A, B, C, and D conditions, the MTCNN model achieves optimization based on the TCNN model. In addition, the MTCNN model was used to further test the fault diagnosis results using different types of signals, as shown in Figure 10.

It can be observed that the MTCNN model has the best performance when using three types of signals. The experiment result indicates that, compared with the TCNN model, the MTCNN model uses the feature extraction ability to extract features with relatively high reliability by setting the weight of multiple types of signals and enhancing processing capability for multiple types of signals. On this basis, try to continue optimizing the fault diagnosis results with the automatic setting of hyperparameters by introducing the hyperparameter optimization module.

### 4.5. Effectiveness Analysis of OMTCNN Model for Pump Fault Diagnosis under Variable Background Condition

**Figure 11:**
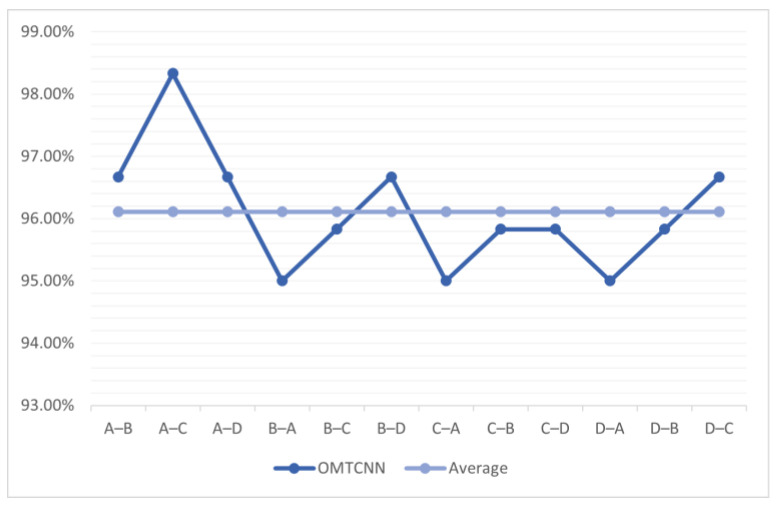
The accuracy of OMTCNN under different background conditions in 1#.

In order to verify the optimization results of fault diagnosis through the introduction of the hyperparameter optimization module, this experiment uses the OMTCNN model to diagnose faults through transfer learning within the 1# cylinder under A, B, C, and D conditions, using any background condition as the source domain and other background conditions as the target domain. The OMTCNN model has added a hyperparameter optimization module on top of the MTCNN model, and its parameter configuration is consistent with the MTCNN model. The accuracy of OMTCNN under A, B, C, and D conditions of internal transfer in a 1# cylinder is shown in Figure 11.

**Figure 12:**
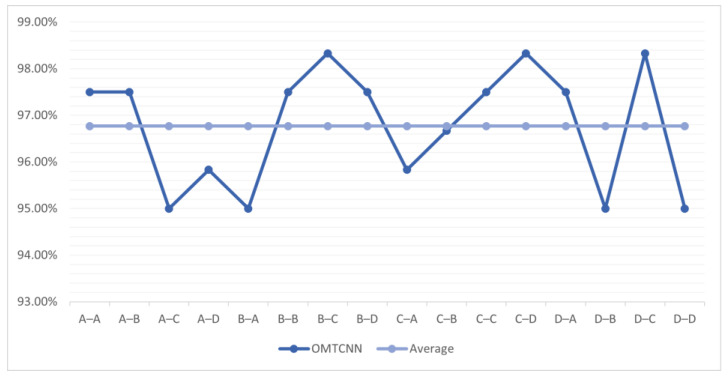
The accuracy of OMTCNN under different background conditions ranges from 2# transfer to 1#.

Further experimentation using the MTCNN model to diagnose faults in the A, B, C, and D conditions of the 1# cylinder through transfer learning using any background condition in the 2# cylinder as the source domain and other background conditions in the 1# cylinder as the target domain The accuracy of the OMTCNN model under A, B, C, and D conditions from 2# transfer to 1# is shown in Figure 12.

It can be observed that, compared to the MTCNN model, the OMTCNN model has higher fault diagnosis accuracy in the internal transfer of the 1# cylinder and the transfer from the 2# cylinder to the 1# cylinder under A, B, C, and D conditions.

In order to explore the performance improvement of the OMTCNN model compared to the MTCNN model, statistical analysis of relevant indicators of OMTCNN and MTCNN is required, as shown in Table 9 and Table 10.

**Figure 13:**
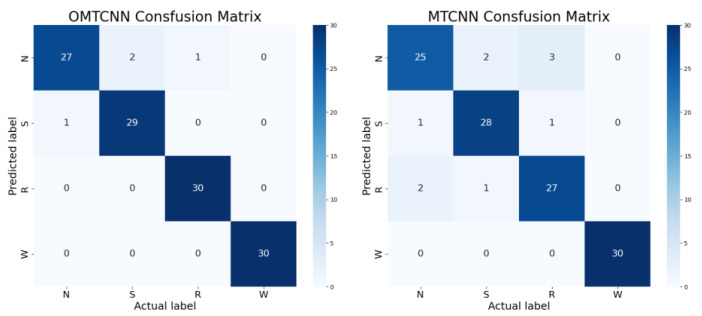
The confusion matrix of OMTCNN and MTCNN.

**Figure 14:**
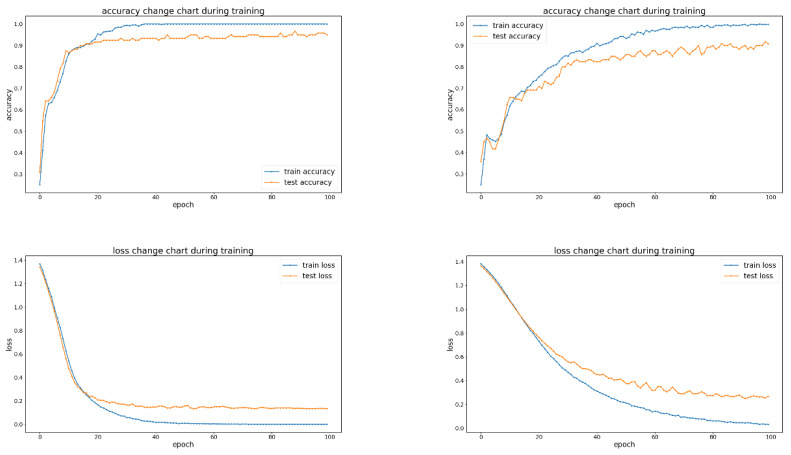
The training process of OMTCNN and MTCNN.

It can be observed that OMTCNN leads MTCNN in relevant statistical indicators. In order to further explore the potential performance improvement of the OMTCNN model compared to the MTCNN model, as shown in Figure 13 and Figure 14, the confusion matrix obtained under the same experimental limitations and the corresponding training process were analyzed. The confusion matrix represents four types of fault status, namely N, S, R, and W. Each fault status contains 30 samples.

According to the confusion matrix analysis, firstly, the diagnostic accuracy of the OMTCNN model is higher than that of the MTCNN model for all fault status types. Secondly, the OMTCNN model has more fault status types with no diagnostic errors than the MTCNN model. Finally, the fault status types that the OMTCNN model experiences misjudgments are strictly within the fault status types that the MTCNN model experiences misjudgments. Reflects the advantages of OMTCNN in terms of diagnostic accuracy.

According to the analysis of the training process, firstly, at the same number of epochs, compared to the MTCNN model, the accuracy of the OMTCNN model is often higher and the Loss is often lower. Secondly, under the same accuracy or Loss, the OMTCNN model often has fewer epochs compared to the MTCNN model. Finally, the OMTCNN model did not exhibit any significant accuracy fallback phenomenon as observed in the MTCNN model. Reflects the advantages of OMTCNN in terms of computational efficiency.

**Figure 15:**
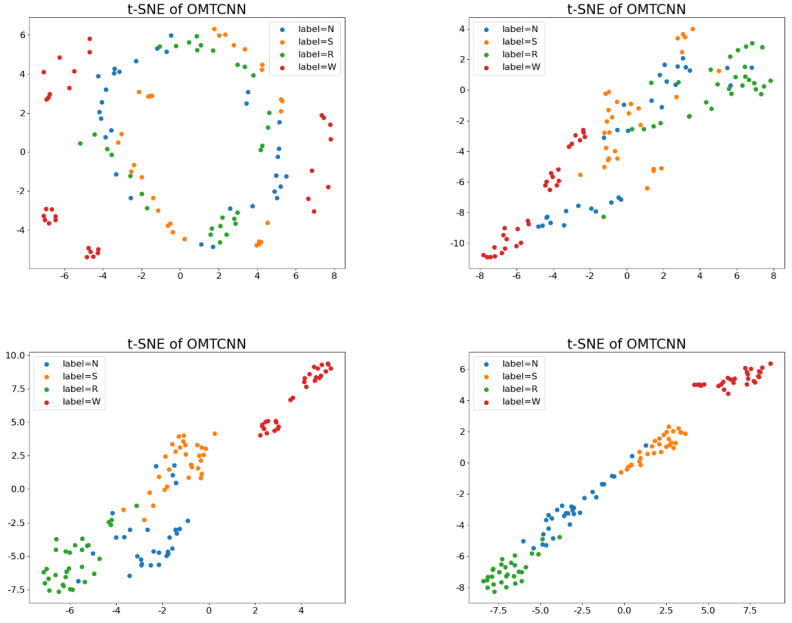
The t-SNE of OMTCNN.

The experiment result indicates that, compared with the MTCNN model, the OMTCNN model improves the ability to extract features by automatically setting the hyperparameters and achieves performance improvement. In addition, the t-SNE of inputs Cov1, Cov2, and Cov3 for describing the performance of the OMTCNN model is provided as shown in Figure 15.

It can be observed that through the OMTCNN model, the characteristics of different fault status types can be clearly separated. Therefore, the OMTCNN model can achieve fault diagnosis for pumps.

## 5. Conclusions

In this article, a novel OMTCNN model is proposed for pump fault diagnosis under different background conditions. First, a new transfer-based convolution neural network model is designed to promote well-learned knowledge transfer over different background conditions for improving robustness and generalizing the model in cross-domain diagnosis tasks so that pump fault diagnosis can be realized under different background conditions. Second, the multi-signal fusion strategy is involved in capturing state information of the pump for establishing the mapping relationship between raw signal and fault pattern by integrating multi-physical signal with weight allocation protocol, thereby implementing fusion processing of multiple types of signals. Finally, a hyperparameter optimization method is explored to align with the transfer learning model by integrating Grid Search with the Gradient Descent algorithm for further improving diagnosis performance and thus achieving optimization of hyperparameter settings. The results show that, compared with other models, the OMTCNN model has excellent performance for pump fault diagnosis and can better realize pump fault diagnosis under different background conditions.

## Figures and Tables

**Table 1:** Parameter configuration of the basic CNN model.

Layer	Kernel Size	Padding	Activate Function	Dropout
Cov1	3 × 3	1 × 1	ReLU	-
Cov2	3 × 3	1 × 1	ReLU	-
Cov3	3 × 3	1 × 1	ReLU	-
MP	2 × 2	-	-	-
FC1	-	-	ReLU	0.2
FC2	-	-	ReLU	0.2
FC3	-	-	Softmax	0.2

**Table 2:** Accuracy of using CNN for 1# and 2# under different background conditions.

1#			2#		
SPM\Mpa	20	40	SPM\Mpa	20	40
90	51.67%	53.33%	90	55.00%	51.67%
110	59.17%	55.83%	110	57.50%	59.17%

**Table 3:** Accuracy of using TCNN for 1# under different background conditions.

A Based Transfer Learning			B Based Transfer Learning		C based Transfer Learning		D Based Transfer Learning	
SPM\Mpa	20	40	20	40	20	40	20	40
90	A	68.33%	71.67%	B	72.50%	70.83%	70.00%	74.17%
110	70.00%	73.33%	70.83%	72.50%	C	67.50%	74.17%	D

**Table 4:** Accuracy of using TCNN for 2# under different background conditions.

A Based Transfer Learning			B Based Transfer Learning		C Based Transfer Learning		D Based Transfer Learning	
SPM\Mpa	20	40	20	40	20	40	20	40
90	A	68.33%	67.50%	B	71.67%	67.50%	68.33%	70.83%
110	74.17%	68.33%	71.67%	67.50%	C	74.17%	71.67%	D

**Table 5:** Accuracy of using TCNN from 2# transfer to 1# under different background conditions.

2#A Based Transfer Learning			2#B Based Transfer Learning		2#C Based Transfer Learning		2#D Based Transfer Learning	
SPM\Mpa	20	40	20	40	20	40	20	40
90	60.83%	60.00%	63.33%	64.17%	65.00%	63.33%	65.00%	60.00%
110	60.83%	60.83%	62.50%	60.83%	63.33%	63.33%	65.83%	66.67%

**Table 6:** Accuracy of using TCNN from 1# transfer to 2# under different background conditions.

1#A Based Transfer Learning			1#B Based Transfer Learning		1#C Based Transfer Learning		1#D Based Transfer Learning	
SPM\Mpa	20	40	20	40	20	40	20	40
90	60.00%	63.33%	64.17%	60.00%	65.00%	63.33%	65.00%	64.17%
110	63.33%	65.00%	63.33%	60.00%	64.17%	61.67%	65.00%	66.67%

**Table 7:** Structure of MTCNN and other methods.

Method	Number of Layers	Conv Kernel Size	Activate Function
MTCNN	7	3 × 3	ReLU
Method1 [34]	8	3 × 1	ReLU
Method2 [35]	7	3 × 3, 3 × 1	ReLU
Method3 [36]	5	3 × 1	Sigmoid

**Table 8:** Computation efficiency of MTCNN and other methods.

Method	Total Params Number	Total Size (MB)	Forward Time (ms)
MTCNN	8386	0.19	1.66
Method1	8436	0.23	1.45
Method2	8176	0.15	1.85
Method3	8376	0.09	0.71

**Table 9:** Statistical indicators of OMTCNN.

	Precision	Recall	F1-Score
N	96.43%	90.00%	93.10%
S	93.55%	96.67%	95.08%
R	96.77%	100%	98.36%
W	100%	100%	100%

**Table 10:** Statistical indicators of MTCNN.

	Precision	Recall	F1-Score
N	89.29%	83.33%	86.21%
S	90.32%	93.33%	91.80%
R	87.10%	90.00%	88.52%
W	100%	100%	100%

## Data Availability

The data are not publicly available due to their containing information that could compromise the privacy of research participants.
